# Enhancing Patient Outcomes: A Novel Nomogram Prediction Model Based on Systemic Immune‐Inflammation Index for Esophageal Stricture After Endoscopic Submucosal Dissection

**DOI:** 10.1002/cam4.70264

**Published:** 2024-09-29

**Authors:** Chen Wang, Mengqiu Tang, Dawei Chen, Yang Zhou, Gaofeng Liang, Ruiwei Shen, Tian Chen

**Affiliations:** ^1^ Department of Gastroenterology, Ningbo Medical Center Lihuili Hospital Ningbo University Ningbo China; ^2^ Department of Radiation Oncology, Ningbo Medical Center Lihuili Hospital Ningbo University Ningbo China; ^3^ Department of Ningbo Institute of Innovation for Combined Medicine and Engineering, Ningbo Medical Center Lihuili Hospital Ningbo University Ningbo China; ^4^ Department of Thoracic Surgery, Ningbo Medical Center Lihuili Hospital Ningbo University Ningbo China

**Keywords:** endoscopic submucosal dissection, esophageal stricture, nomogram model, systemic immune‐inflammation index, treatment outcomes

## Abstract

**Background:**

Endoscopic submucosal dissection (ESD) is a widely utilized treatment for early esophageal cancer. However, the rising incidence of postoperative esophageal stricture poses a significant challenge, adversely affecting patients' quality of life and treatment outcomes. Developing precise predictive models is urgently required to enhance treatment outcomes.

**Materials and Methods:**

This study retrospectively analyzed clinical data from 124 patients with early esophageal cancer who underwent ESD at Ningbo Medical Center Lihuili Hospital. Patients were followed up to assess esophageal stricture incidence. Binary logistic regression analysis was used to identify factors associated with post‐ESD esophageal stricture. A novel nomogram prediction model based on Systemic Immune‐inflammation Index (SII) was constructed and evaluated using receiver operating characteristic (ROC) curves, calibration curves, and decision curve analysis (DCA).

**Results:**

ROC curve analysis showed that the optimal value of SII for predicting esophageal stricture was 312.67. Both univariate and multivariate analyses identified lesion infiltration depth (< M2 vs. ≥ M2, *p* = 0.002), lesion longitudinal length (< 4 cm vs. ≥ 4 cm, *p* = 0.008), circumferential resection range (< 0.5, 0.5–0.75, ≥ 0.75, *p* = 0.014), and SII (< 312.67 vs. ≥ 312.67, *p* = 0.040) as independent risk factors for post‐ESD esophageal stricture. A novel nomogram prediction model incorporating these four risk factors was developed. Validation using ROC curve analysis demonstrated satisfactory model performance, while calibration curves indicated good agreement between model‐predicted risk and observed outcomes.

**Conclusion:**

We successfully constructed a novel nomogram prediction model based on SII, which can accurately and intuitively predict the occurrence of esophageal stricture after ESD, providing guidance for clinicians and improving treatment outcomes.

## 1 Background

Esophageal cancer ranks among the most prevalent malignancies globally, with a particularly notable incidence in China [1]. Despite advancements in early detection and treatment, endoscopic submucosal dissection (ESD) has emerged as a widely adopted therapeutic approach. However, the escalating use of ESD has brought to light a rising occurrence of postoperative esophageal stricture, reaching rates as high as 30% in certain cohort studies [2, 3, 4]. Postoperative esophageal stricture can manifest with symptoms such as dysphagia, malnutrition, and bleeding, escalating treatment costs and recovery periods, and profoundly impacting patients' quality of life and treatment outcomes [2, 5]. Therefore, accurate prediction of postoperative esophageal stricture occurrence holds paramount clinical significance.

Various biomarkers and models have been proposed for predicting postoperative esophageal stricture, primarily focusing on intraoperative lesion characteristics [2, 3]. These factors often consider external influences while overlooking intrinsic physiological factors, limiting their clinical utility. Post‐ESD esophageal stricture primarily ensues from inflammation and fibrous tissue hyperplasia [6, 7]. To date, only two studies have investigated the predictive value of inflammatory markers, such as neutrophil‐lymphocyte ratio (NLR), platelet‐lymphocyte ratio (PLR), and monocyte‐lymphocyte ratio (MLR), and demonstrated the predictive ability of NLR and MLR in post‐ESD esophageal stricture [8, 9]. The systemic immune‐inflammation index (SII), encompassing platelets, neutrophils, and lymphocytes, offers a comprehensive reflection of a patient's inflammatory and immune status and may surpass NLR, PLR, and MLR in predictive accuracy [10, 11, 12]. Integrating SII into the prediction of esophageal stricture after esophageal cancer ESD could enhance prediction accuracy and reliability, providing novel insights and evidence for personalized treatment strategies.

To more intuitively demonstrate the role of the SII in predicting esophageal stricture after esophageal cancer ESD, we not only retrospectively analyzed factors associated with post‐ESD esophageal stricture in patients with early esophageal cancer but also devised a prediction model grounded on SII and converted it into an intuitive prediction nomogram, providing guidance for clinical decision‐making and enhancing the quality of life and treatment outcomes for patients.

## 2 Materials and Methods

### 2.1 Patients

This study retrospectively collected data from patients diagnosed with early esophageal cancer who underwent ESD at Ningbo Medical Center Lihuili Hospital between January 2015 and January 2023. Inclusion criteria were as follows: (1) age 18 years or older, (2) histologically confirmed low‐grade intraepithelial neoplasia (LGIN), high‐grade intraepithelial neoplasia (HGIN), or esophageal squamous cell carcinoma, (3) lesion infiltration depth not exceeding 200 μm submucosally, (4) no lymph node metastasis, (5) blood routine values obtained within 3 days before ESD. Exclusion criteria were as follows: (1) additional chemotherapy or surgical treatment after ESD, (2) simultaneous ESD for multiple lesions in the esophagus, (3) concomitant other tumors, rheumatic immune diseases, or diabetes mellitus, (4) long‐term oral corticosteroid or immunosuppressive therapy before ESD, (5) infectious diseases within the past half month, (6) loss to follow‐up or death within 1 year for any reason.

The ESD procedures were executed by a team of four highly skilled surgeons, each with substantial expertise in gastrointestinal endoscopy and a proven record of surgical success. To ensure uniformity in the approach, standardized single‐channel endoscopes (GIF‐H260, Olympus, Tokyo, Japan) were used consistently across all procedures. For optimal visualization and precise dissection, a short transparent cap (ND‐201‐11,802; Olympus) was attached to the gastroscope's tip, facilitating a consistent endoscopic view. The boundaries of the lesions were demarcated using Argon Plasma Coagulation, providing a clearly defined resection area. Submucosal elevation of the lesions was achieved by injecting a saline solution containing epinephrine at a concentration of 1:10,000, which aided in dissection while reducing the risk of perforation. Lesions were circumferentially incised and completely excised using a Dual knife, ensuring consistent application of cutting and coagulation currents. Hemostasis was meticulously managed with thermal biopsy forceps, further standardizing the procedure across cases. At our center, rigorous internal quality control measures are implemented to ensure procedural consistency and surgical precision. These measures include the use of standardized reporting forms for each step of the ESD procedure and a detailed review of the endoscopic images captured during the operation.

The study was conducted following the principles of the Declaration of Helsinki. The Ningbo Medical Center Lihuili Hospital Ethics Committee approved this retrospective study, and the requirement for informed patient consent was waived (KY2024SL205–01).

### 2.2 Data Collection and Follow‐Up

Clinical data, encompassing gender, age, lesion characteristics, preoperative blood routine data, and post‐ESD esophageal stricture occurrence, were gathered for all enrolled patients. The SII was defined as neutrophil count × platelet count/lymphocyte count. Esophageal stricture was defined as the presence of dysphagia symptoms or inability to pass a standard endoscope (diameter 9.8 mm) [13]. Regular gastroscopy evaluations were performed at 1, 6, and 12 months after ESD, or immediately if obstructive symptoms occurred postoperatively.

### 2.3 Statistical Methods

Data analysis was conducted using SPSS statistical software (version 26.0, IBM) and R version 4.0.1 (R Foundation for Statistical Computing). The optimal cutoff value of the SII for predicting esophageal stricture was determined using receiver operating characteristic (ROC) curve analysis. Optimal cutoff values for other clinical features were selected based on recognized values, such as the infiltration depth cutoff at M2 and the lesion longitudinal length cutoff at 4 cm. Additionally, based on the circumferential extent of the post‐ESD mucosal defect relative to the luminal circumference, patients were stratified into three distinct groups using the clock face method: < 0.5, 0.5–0.75, and ≥ 0.75 [2]. Chi‐squared tests were used to analyze categorical variables. Binary logistic regression analysis was performed to identify factors associated with esophageal stricture. Finally, a nomogram prediction model based on the SII was constructed, and the model was evaluated using ROC curves, calibration curves, and decision curve analysis (DCA). A two‐sided *p* < 0.05 was considered statistically significant.

## 3 Results

### 3.1 Patient Characteristics

The study enrolled 124 patients diagnosed with early esophageal cancer, with males constituting 112 cases (90.3%) and a median age of 63 years (range 43–80 years). Esophageal lesion locations were mainly distributed in the middle and lower segments, accounting for 113 cases (91.1%). The majority of patients had lesions with infiltration depth ≥ M2, accounting for 90 cases (72.6%). Fourteen patients (12.6%) had a circumferential resection range exceeding three‐quarters. 11 of these patients were administered a standardized 8‐week short‐course oral prednisone regimen commencing postoperatively. The remaining 3 patients did not receive this specific therapeutic intervention. Patients with a circumferential resection range less than three‐quarters did not receive special treatment to prevent esophageal stricture. During the follow‐up period, 41 patients (33.1%) experienced esophageal stricture. Detailed information regarding patient demographics and pathological characteristics is presented in Table 1.

**TABLE 1:** Fundamental patient characteristics and univariate analysis for esophageal stricture.

Characteristics	Non‐stricture (%)	Stricture (%)	*p*
Age (years)			
≤ 60	39 (31.5%)	15 (12.1%)	0.272
> 60	44 (35.5%)	26 (20.9%)	
Gender			
Female	10 (8.1%)	2 (1.6%)	0.334
Male	73 (58.9%)	39 (31.4%)	
Drinking			
No	24 (19.4%)	14 (11.3%)	0.552
Yes	59 (47.6%)	27 (21.7%)	
Smoking			
No	32 (25.8%)	17 (13.7%)	0.755
Yes	51 (41.1%)	24 (19.4%)	
Lesion location			
Cervical	1 (0.8%)	3 (2.4%)	0.149
Upper	3 (2.4%)	4 (3.2%)	
Middle	31 (25.0%)	15 (12.1%)	
Lower	48 (38.7%)	19 (15.4%)	
Pathological type			
Precancerous lesions	41 (33.1%)	14 (11.3%)	0.108
Squamous cell carcinoma	42 (33.9%)	27 (21.7%)	
Paris type			
IIa	15 (10.8%)	9 (4.5%)	0.115
IIb	60 (51.4%)	23 (20.7%)	
IIc	8 (5.4%)	9 (7.2%)	
Circumferential resection range			
< 1/2	66 (53.2%)	15 (12.1%)	0.001
1/2–3/4	12 (9.7%)	17 (13.7%)	
> 3/4	5 (4.0%)	9 (7.3%)	
Longitudinal length (cm)			
< 4	60 (48.4%)	13 (10.5%)	0.001
≥ 4	23 (18.5%)	28 (22.6%)	
Infiltration depth			
< M2	33 (26.6%)	1 (0.8%)	0.001
≥ M2	50 (40.3%)	40 (32.3%)	
SII			
< 312.67	40 (32.3%)	11 (8.9%)	0.023
≥ 312.67	43 (34.7%)	30 (24.1%)	

Abbreviations: M2, muscularis mucosa; SII, systemic immune‐inflammation index.

### 3.2 Optimal Cutoff Value of the SII

**FIGURE 1:**
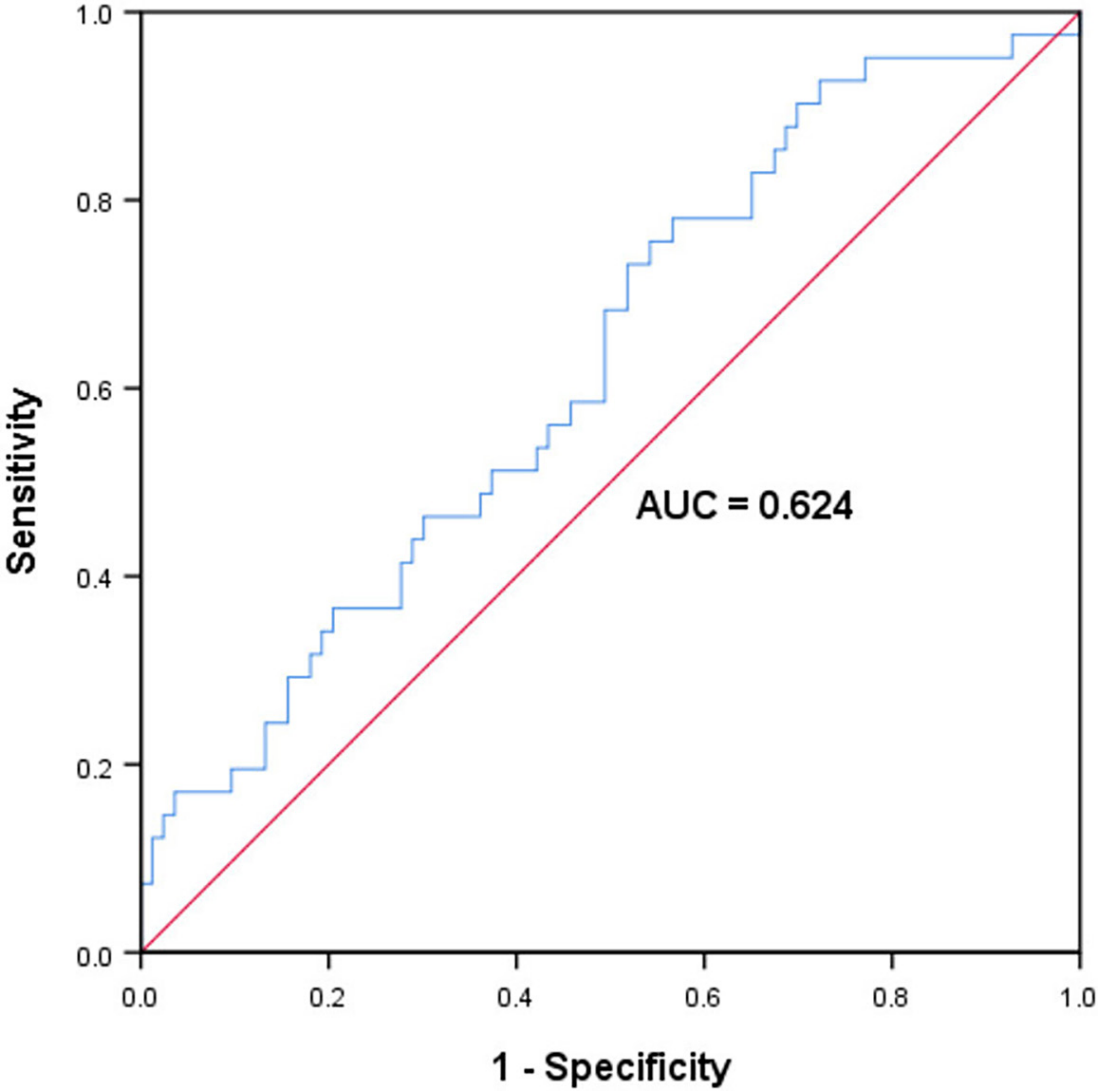
Receiver operating curves for esophageal stricture showing the optimum cutoff values for Immune‐inflammation Index (SII).

The median value of the SII was 360.21 (33.68–1355.20). The ROC curve analysis revealed the optimal SII cutoff value for predicting esophageal stricture to be 312.67, with an AUC of 0.624 (95% CI 0.521–0.727, *p* = 0.026), sensitivity of 73.2%, and specificity of 51.8% (Figure 1).

### 3.3 Risk Factors for Post‐ESD Esophageal Stricture

Univariate analysis demonstrated significant associations between post‐ESD esophageal stricture and factors including lesion infiltration depth (< M2 vs. ≥ M2, *p* = 0.001), lesion longitudinal length (< 4 cm vs. ≥ 4 cm, *p* = 0.001), circumferential resection range (< 0.5, 0.5–0.75, ≥ 0.75, *p* = 0.001), and SII (< 312.67 vs. ≥ 312.67, *p* = 0.023). Other factors such as lesion location (*p* = 0.149) and pathological type (*p* = 0.108) were found to be unrelated to esophageal stricture (Table 1). Furthermore, factors with *p* < 0.1 in the univariate analysis were incorporated into the multivariable logistic regression analysis, resulting in four factors: lesion infiltration depth, lesion longitudinal length, circumferential resection range, and SII. The results indicated that lesion infiltration depth (*p* = 0.002), lesion longitudinal length (*p* = 0.008), circumferential resection range (*p* = 0.014), and SII (*p* = 0.040) remained independent risk factors (Table 2).

**TABLE 2:** Multivariate logistic regression model.

Characteristics	Esophageal stricture		
	RR	95% CI	*p*
Circumferential resection range			0.014
< 1/2	1		
1/2–3/4	3.818	1.209–12.063	0.022
> 3/4	5.857	1.419–24.173	0.015
Longitudinal length			
< 4 cm	1		
≥ 4 cm	3.794	1.419–10.142	0.008
Infiltration depth			
< M2	1		
≥ M2	27.619	3.218–237.024	0.002
SII			
< 312.67	1		
≥ 312.67	2.956	1.053–8.303	0.040

Abbreviations: CI, confidence interval; M2, muscularis mucosa; RR, relative risk; SII, systemic immune‐inflammation index.

### 3.4 Construction of the Nomogram

**FIGURE 2:**
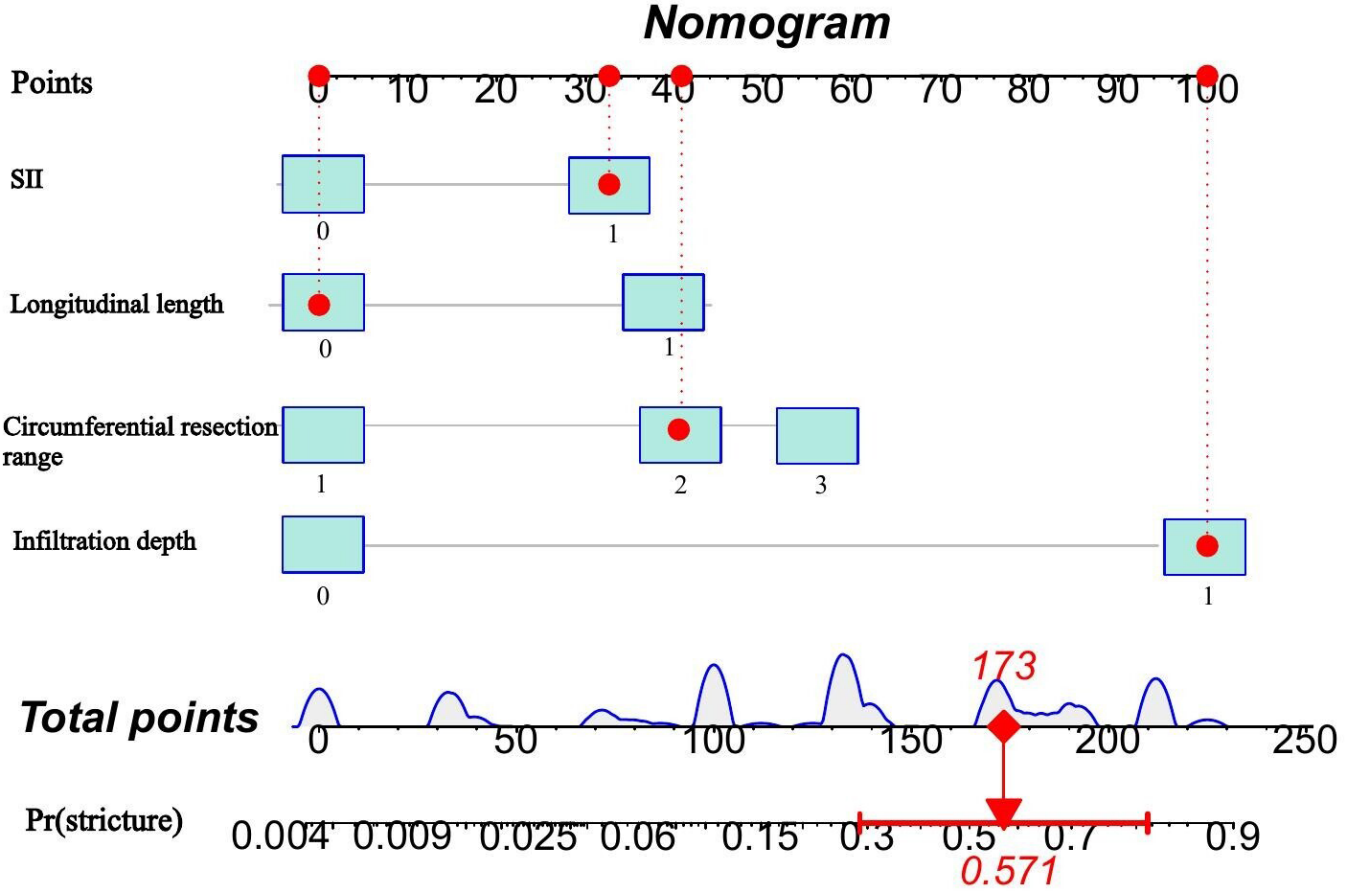
A novel prediction nomogram of esophageal stricture for patients undergoing endoscopic submucosal dissection.

A multicollinearity diagnostic analysis was performed on the aforementioned independent risk factors (lesion infiltration depth, lesion longitudinal length, circumferential resection range, SII), revealing relatively low correlations among them. By calculating the variance inflation factor (VIF), the results showed VIF values of 1.043, 1.177, 1.179, and 1.014 for each factor, with tolerances greater than 0.8, indicating no multicollinearity among these four independent risk factors. Based on the results of the multifactorial analysis, we developed a novel nomogram prediction model integrating SII with other independent risk factors (Figure 2).

### 3.5 Nomogram Validation

**FIGURE 3:**
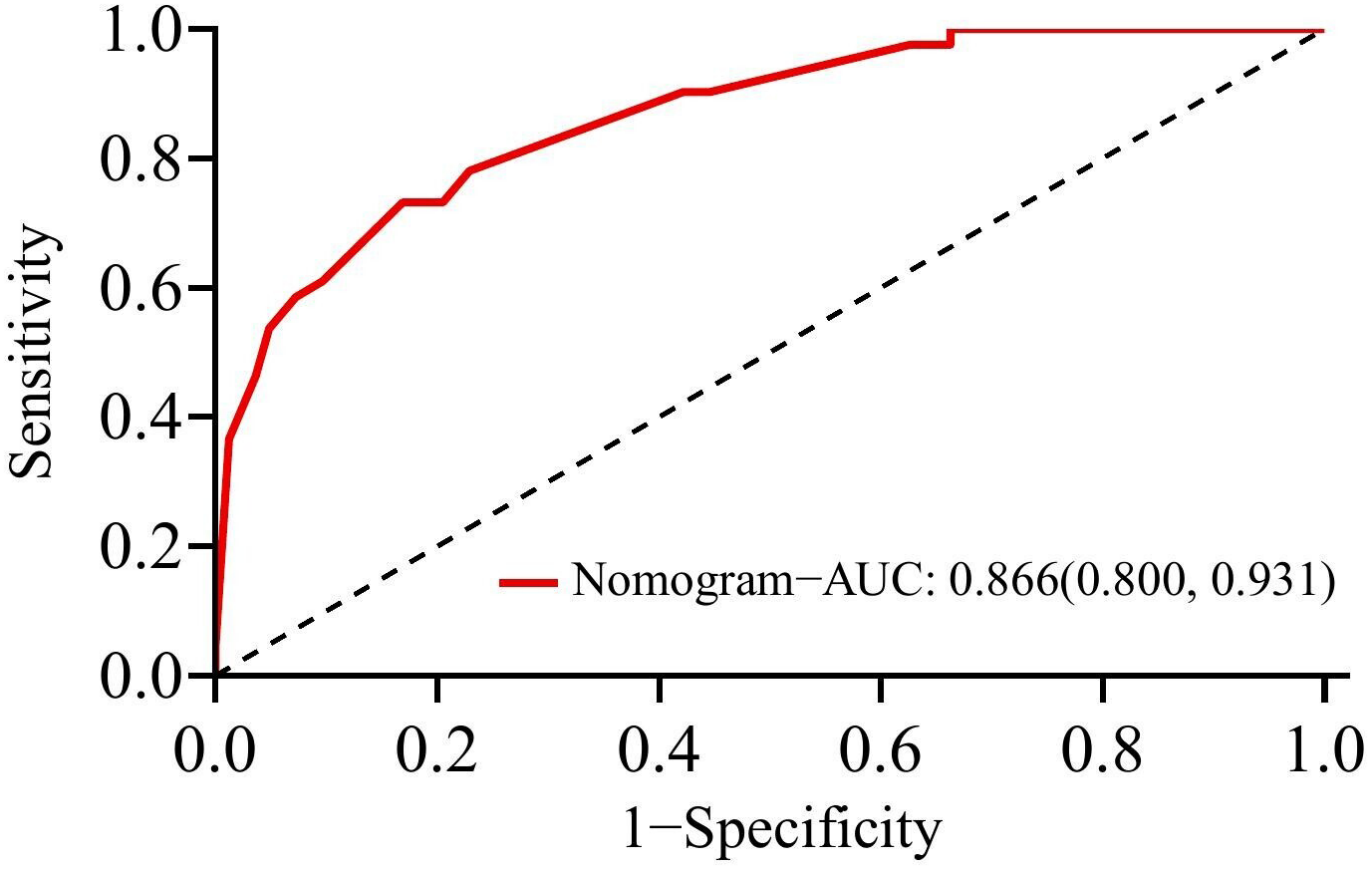
Receiver operating characteristic curves for the novel nomogram prediction model based on Immune‐inflammation Index (SII).

**FIGURE 4:**
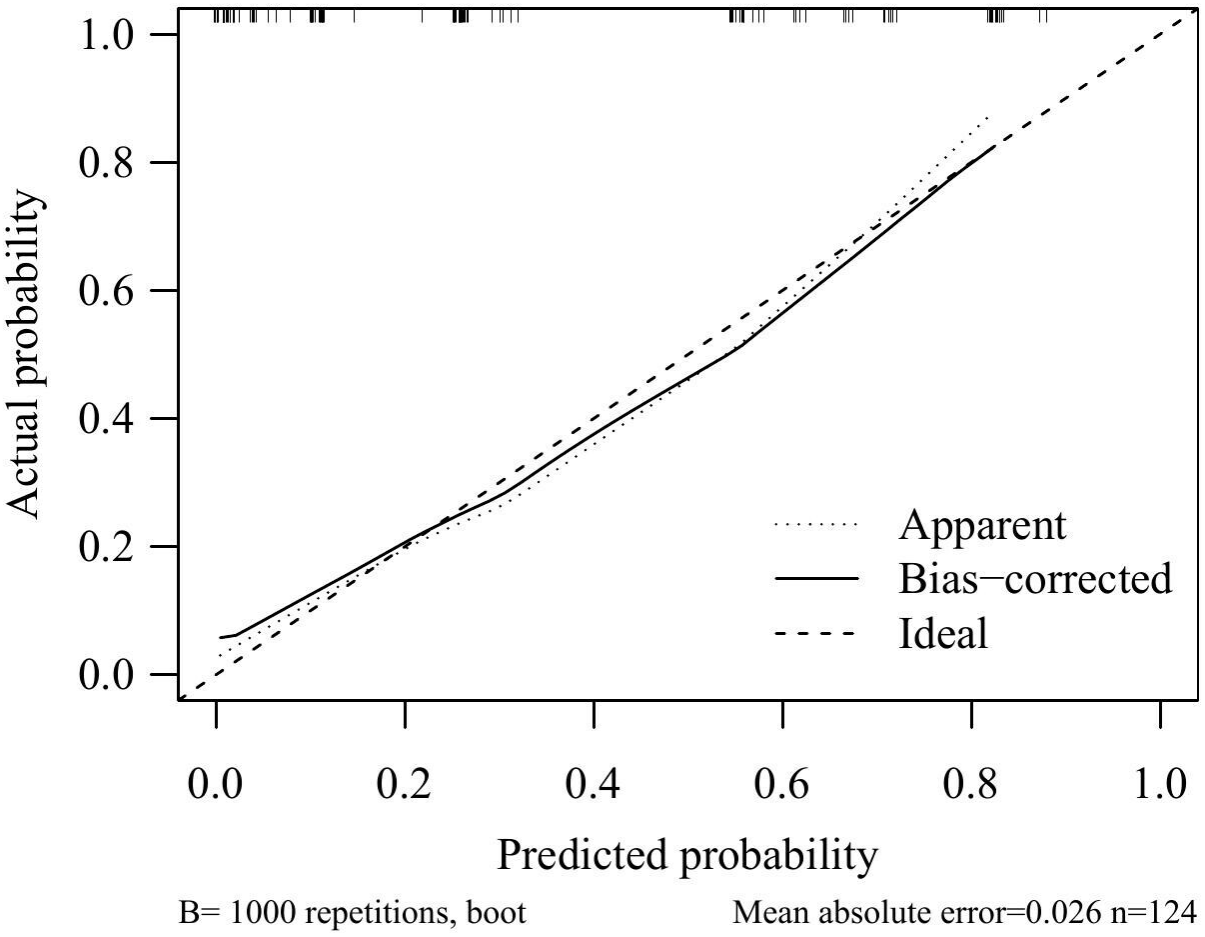
Calibration curves for the test accuracy of the novel nomogram prediction model.

**FIGURE 5:**
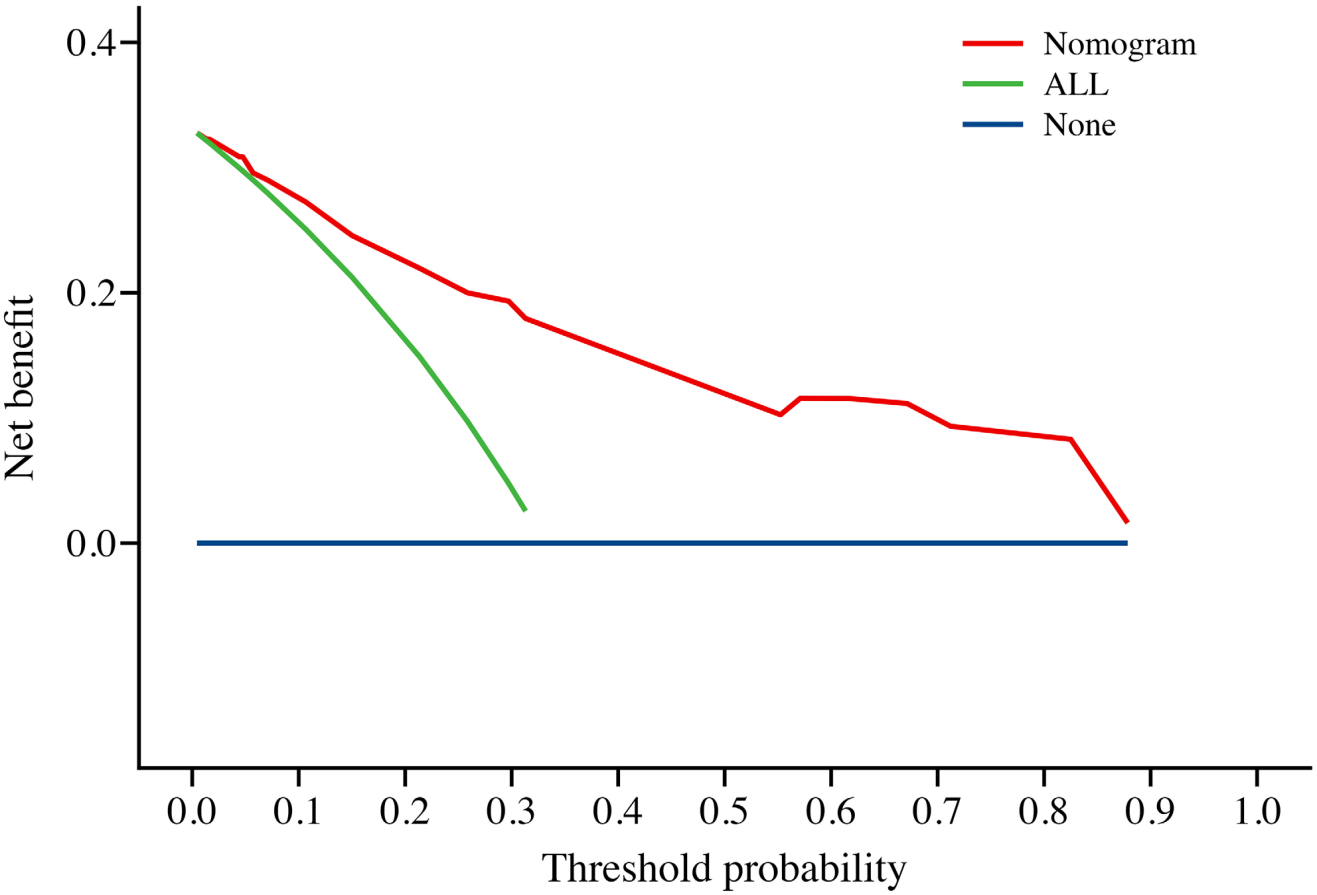
Decision curve analysis of the novel nomogram prediction model.

The performance of the novel nomogram prediction model based on SII was validated by plotting the ROC curve (Figure 3), yielding an AUC of 86.6%, with sensitivity and specificity of 73.2% and 83.1%, respectively, indicating excellent discriminative and predictive capabilities. The calibration curve (Figure 4) demonstrated good consistency between the risk estimates of the prediction model and actual observations. Finally, by quantifying the net benefit at different threshold probabilities, Decision Curve Analysis (DCA) was utilized to assess the clinical utility of the prediction model. DCA revealed that within the threshold probability range of 31% to 88%, utilizing this model for clinical decision‐making could provide higher net benefit (Figure 5).

## 4 Discussion

The widespread use of ESD in treating early esophageal cancer has brought postoperative esophageal stricture to the forefront as a common complication. Previous studies have reported an incidence rate of esophageal stricture ranging from 10% to 30% [2, 3, 4]. The incidence rate of esophageal stricture in this study was 33.1%, which is relatively high compared to previous studies. This high incidence may be influenced by various factors, including the demographics of the patient population and differences in treatment protocols. Since this study involved a single medical center with a relatively small sample size, differences in demographic characteristics of patients, treatment protocols, and medical standards across research centers may contribute to variations in esophageal stricture incidence rates. The majority of patients in this study had an infiltration depth of M ≥ 2, an independent risk factor for esophageal stricture [2, 14], suggesting a higher stricture incidence rate in this group compared to other research centers. Additionally, three patients in this study with over three‐quarters of the circumference removed did not receive oral corticosteroid treatment after ESD, despite evidence showing its efficacy in preventing esophageal stricture [15, 16, 17]. The high incidence rate of esophageal stricture post‐ESD highlights its clinical significance. Thus, accurately predicting postoperative esophageal stricture occurrence is crucial for guiding clinical treatment, reducing complications, and improving patient quality of life. The findings of this study not only further emphasize the high incidence rate of postoperative esophageal stricture but also provide clinicians with an opportunity for timely intervention. By establishing a predictive model, clinicians can identify high‐risk patients postoperatively and take appropriate preventive measures, such as early esophageal dilation or administration of oral corticosteroids, to mitigate complications, enhance treatment outcomes, and improve patient quality of life.

Most established predictive models for esophageal stricture focus on intraoperative lesion characteristics and other external factors. Previous studies consistently indicated that the lesion infiltration depth, longitudinal length, and circumferential resection range are independent risk factors for predicting post‐ESD esophageal stricture [2, 3]. This study's results further confirmed these factors as independent predictors of esophageal stricture. The mechanism of esophageal stricture post‐ESD, considered a wound healing process where inflammatory response plays a crucial role [6, 7, 18], lacks comprehensive understanding, especially regarding intrinsic inflammatory factors' relationship with esophageal stricture prediction. So far, only two studies have analyzed inflammatory markers, with one showing that NLR, PLR, and MLR have predictive value for esophageal stricture and combining these three markers showing better predictive ability [8]. Another study further integrated NLR with lesion‐related factors and successfully established a model for predicting esophageal stricture occurrence [9]. The SII comprehensively considers platelets, neutrophils, and lymphocytes, combining aspects of NLR and PLR, thus providing a more comprehensive reflection of the patient's inflammatory status. Therefore, the SII index is more comprehensive and can more accurately reflect the patient's inflammatory status, thereby improving the accuracy and reliability of prediction. Hence, in order to further investigate potential predictive factors, we intend to evaluate the predictive efficacy of the Prognostic Nutritional Index (PNI), C‐reactive protein to albumin ratio (CAR), and SII in distinct research articles focusing on the development of esophageal stricture following esophageal ESD. One justification for the exclusion of PNI and CAR variables from our analysis stems from the retrospective nature of our study and the limited sample size of patients with concurrent data on PNI, CAR, and SII, which impeded our ability to conduct a comprehensive analysis and modeling. This study's results, for the first time, demonstrate SII as an independent predictor of esophageal stricture. By incorporating SII into the predictive model, both tumor characteristics and the body's inflammatory response are considered, improving predictive accuracy and comprehensiveness. Nomograms, widely used in clinical practice to integrate multiple factors and visually present event probabilities, offer a comprehensive approach to prediction [19, 20]. Therefore, this study establishes a comprehensive nomogram prediction model combining SII with lesion characteristics, intuitively presenting various predictive factors in one chart, aiding clinicians in timely identification of high‐risk patients and implementation of effective preventive measures to reduce esophageal stricture incidence, improve patient prognosis, and enhance quality of life. Finally, DCA confirmed that using the SII‐based nomogram prediction model for clinical decision‐making can provide higher net benefit.

Despite efforts to include early esophageal cancer patients meeting criteria, limitations of this single‐center retrospective design, small sample size, incomplete information retrieval, and uncertain data quality may lead to biases, affecting the stability and reliability of the model. Second, this study did not consider all factors related to esophageal stricture, focusing only on SII as an inflammatory factor, which may limit conclusions. Furthermore, while our research has demonstrated a high level of procedural standardization, we acknowledge the limitations inherent in assessing the consistency among different operators without a formal Kappa consistency analysis. Finally, the model in this study lacks internal and external validation, reducing its reliability and effectiveness. Despite these limitations, this study is the first to identify SII as an independent factor for post‐ESD esophageal stricture and successfully construct a novel nomogram prediction model based on SII. These findings offer a new perspective and method for predicting post‐ESD esophageal stricture in early esophageal cancer patients, providing clinicians with a more comprehensive and accurate risk assessment tool. Future research should involve multicenter, large‐sample prospective studies, along with internal and external validation of predictive models, to ensure their stability, reliability, and widespread applicability.

## 5 Conclusions

SII has been confirmed as an independent predictor of post‐ESD esophageal stricture in early esophageal cancer. Based on this finding, we have successfully developed a novel nomogram prediction model capable of accurately and intuitively predicting the occurrence of esophageal stricture. This model holds the potential to guide clinicians, thereby improving treatment outcomes and enhancing patients' quality of life.

## Author Contributions

**Chen Wang:** writing – original draft (lead), writing – review and editing (equal). **Mengqiu Tang:** writing – original draft (supporting). **Dawei Chen:** data curation (equal), formal analysis (equal). **Yang Zhou:** data curation (equal), formal analysis (equal). **Gaofeng Liang:** data curation (equal), formal analysis (equal). **Ruiwei Shen:** conceptualization (equal). **Tian Chen:** conceptualization (equal), writing – review and editing (equal).

## Ethics Statement

The Ningbo Medical Center Lihuili Hospital Ethics Committee approved this retrospective study, and the requirement for informed patient consent was waived (KY2024SL205‐01).

## Consent

All authors have read this manuscript and agree to publish.

## Conflicts of Interest

The authors declare no conflicts of interest.

## Data Availability

The data used to support the findings of this study are available from the corresponding author upon request.
